# Supplementary material for: Nickel Intercalation in Epitaxial Graphene on SiC(0001): A Novel Platform for Engineering Two‐Dimensional Heterostructures
Source: Adv Sci (Weinh). 2026 Sep 3:e77456. Online ahead of print. doi: 10.1002/advs.77456 (PMC13542409; doi:10.1002/advs.77456)
Supplement: Supplementary file 1 [file ADVS-9999-e77456-s001.pdf]

Supplementary Information for

Nickel intercalation in epitaxial graphene on SiC(0001): a novel platform for engineering two-dimensional heterostructures

Ylea Vlamidis,[1,2] Stiven Forti,[3] Antonio Rossi,[3,4] Arrigo Calzolari,[5] Carmela Marinelli,[2] Camilla Coletti,[3,4] Stefan Heun,[1] and Stefano Veronesi[1]

[1)] *Istituto Nanoscienze–CNR, NEST-Scuola Normale Superiore, Piazza San Silvestro 12, 56127 Pisa, Italy*

[2)] *Department of Physical Science, Earth, and Environment, University of Siena, Via Roma 56, 53100 Siena, Italy*

[3)] *Center for Nanotechnology Innovation@NEST, Istituto Italiano di Tecnologia, Piazza San Silvestro 12, Pisa 56127, Italy*

[4)] *Graphene Laboratories, Istituto Italiano di Tecnologia, Genova 16163, Italy*

[5)] *Istituto Nanoscienze-CNR, S3, Via Campi 213/a, Modena, 40125, Italy*

(Dated: 22 April 2026)

S1. RAMAN ANALYSIS OF EPITAXIAL GRAPHENE

The homogeneity and composition of the graphene was analyzed using Raman spectroscopy. Raman mapping (21x21 μm^2) was carried out with a Renishaw inVia system, utilizing a 100x objective (NA 0.85) and a 532 nm laser. Measurements were taken with an 1800 mm/l grating and a fluence of approximately 35 mJ/μm^2. The Raman spectrum of EG (Figure S1a) exhibits the characteristic G and 2D bands of graphene, centred at 1597 cm^{-1} and 2725 cm^{-1}, respectively. The 2D band shows a full width at half maximum (FWHM) ranging from 30 to 50 cm^{-1}, with an average value of 37.1 cm^{-1} (Figure S1b) and is located between 2715 cm^{-1} and 2735 cm^{-1} (Figure S1d). These characteristics, together with the 2D peak intensity map (Figure S1c), are consistent with the coexistence of monolayer graphene and buffer layer regions. Specifically, the 2D intensity map differentiates areas where the 2D peak is negligible, indicative of the buffer layer, from areas displaying a pronounced 2D peak, characteristic of monolayer graphene.[1] The low intensity of the D band, which is associated with structural defects, further confirms the high crystalline quality of the EG. Additionally, peaks observed at 1520 cm^{-1} and 1715 cm^{-1} are attributed to the SiC substrate and correspond to the overtone of the TO phonon and a combination of optical phonons with wave vectors near the M point at the Brillouin zone edges, respectively.[2]

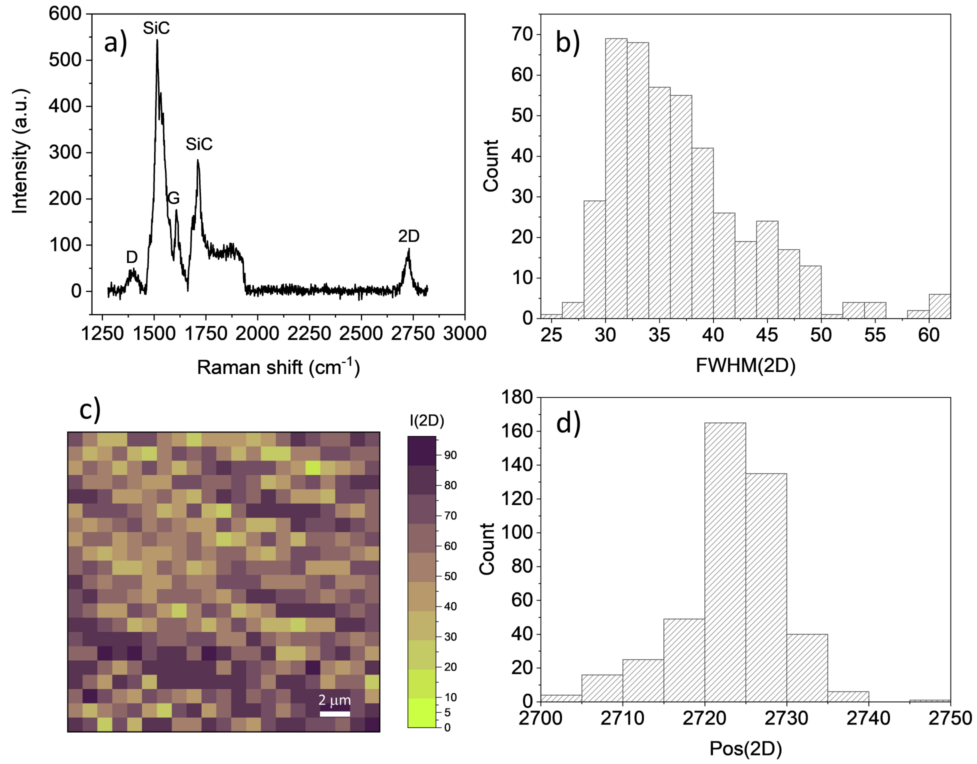

FIG. S1. Raman analysis of EG grown on 6H-SiC(0001) extracted from a $21 \times 21\ \mu m^2$ map: (a) Representative Raman spectrum, (b, d) Histograms displaying the distribution of Raman parameters, specifically (b) the full width at half maximum (FWHM) of the 2D band and (d) the 2D band position for graphene. (c) Raman intensity map of the 2D band.

S2. AFM CHARACTERIZATION OF PRISTINE AND Ni NPs-DECORATED EPITAXIAL GRAPHENE

The surface morphology of EG samples was investigated both before and after functionalization with Ni NPs using atomic force microscopy (AFM, Bruker Dimension Icon) in tapping mode. The scans were performed at a rate of 1 Hz with 512 scanning lines. As shown in Figure S2a, EG on SiC comprises large monolayer graphene regions alongside a stripe-patterned buffer layer. Figure S2b highlights the effect of Ni NPs deposition (nominal size 10 nm) on this surface, achieved by immersing the EG sample in a water/ethanol dispersion (0.05 mg mL^{-1}). The resulting nanoparticle distribution appears uniform, forming a dense, snowflake-like pattern with evident clusters with diameters ranging from 100 to 200 nm, though without any discernible long-range ordering on the surface.

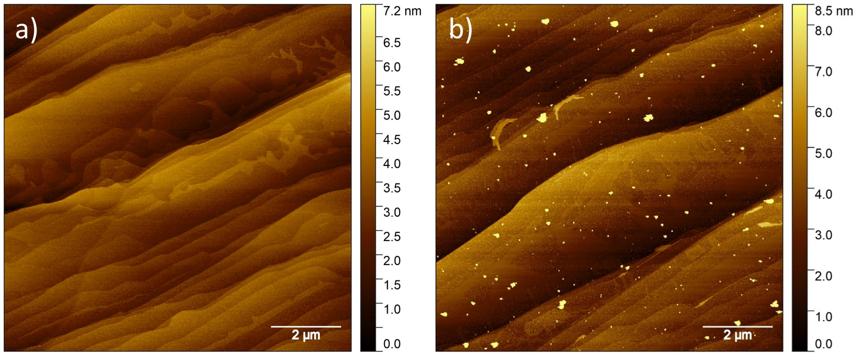

FIG. S2. AFM topographies (10×10 μm^2): (a) pristine EG sample grown on 6H-SiC (0001), and (b) after it surface functionalization with Ni NPs.

S3. FURTHER MICROSCOPY DATA

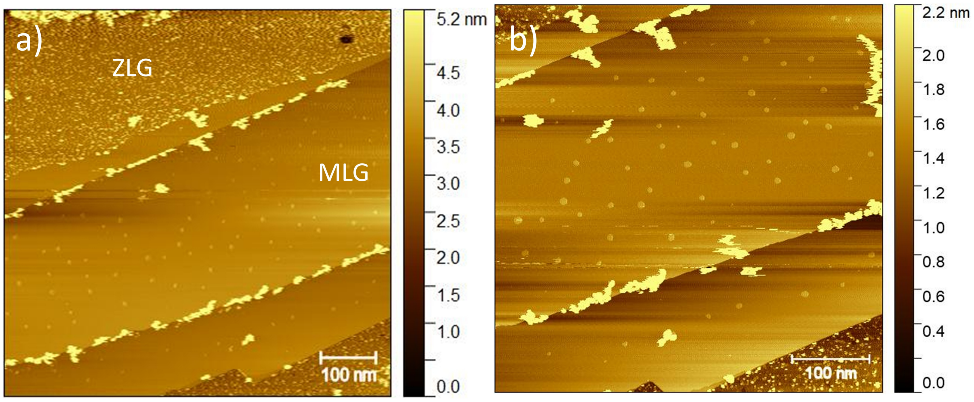

FIG. S3. STM topographies of Ni islands intercalated in EG on 6H-SiC(0001), after annealing at 650°C for 2 minutes. (a) 700 × 700 nm^2 area acquired in a ZLG/MLG region, and (b) a 200 × 200 nm^2 area in a MLG region. The images reveal clusters of nanoparticles located along the step edges and a random distribution of intercalated Ni islands within the MLG regions. Imaging parameters: (a) V_s= 0.6 V, I_t = 0.50 nA, (b) V_s= 0.4 V, I_t = 0.30 nA.

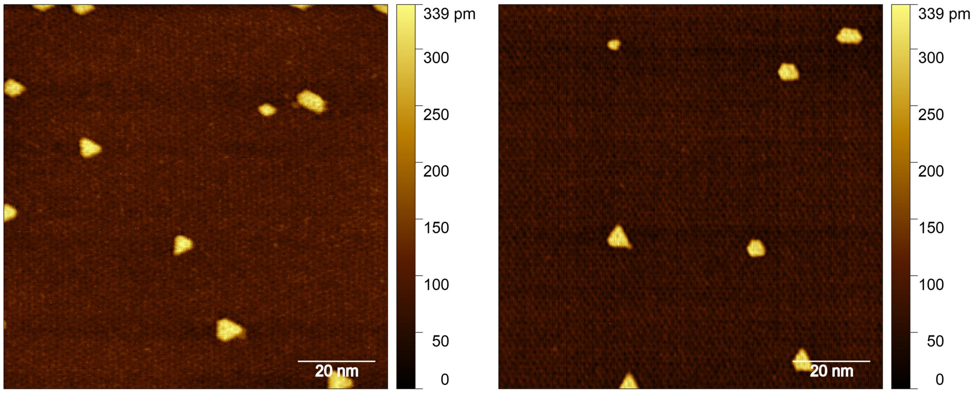

FIG. S4. STM topography images (100 × 100 nm^2) of Ni islands intercalated in EG on 6H-SiC(0001) acquired (a) immediately after Ni intercalation and (b) after exposure of the sample to ambient conditions, followed by reintroduction into UHV. Imaging parameters: V_s= 0.8 V, I_t = 0.70 nA.

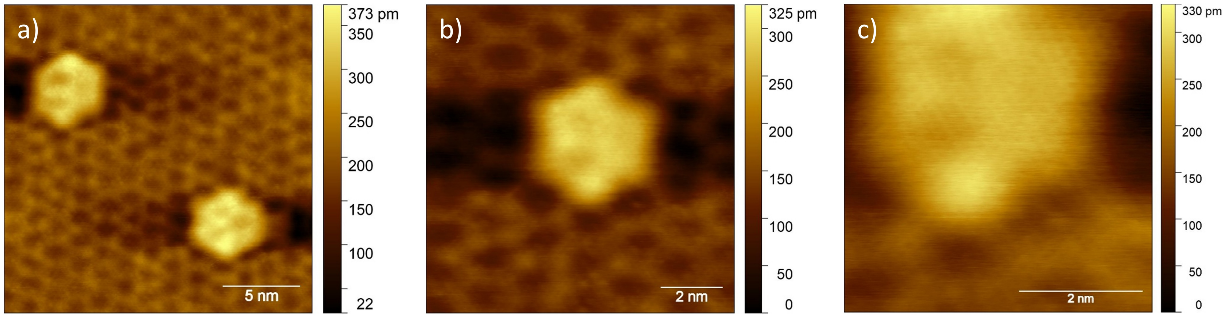

FIG. S5. STM images of Ni islands intercalated in EG on 6H-SiC(0001), after annealing at 650°C for 2 minutes. (a) Image 20 × 20 nm^2, (b) expanded view of 10 × 10 nm^2, and (c) expanded view of 5 × 5 nm^2. Imaging parameters: (a) V_s= 0.4 V, I_t = 0.30 nA; (b) V_s= 0.35 V, I_t = 0.30 nA; (c) V_s= 0.3 V, I_t = 0.25 nA.

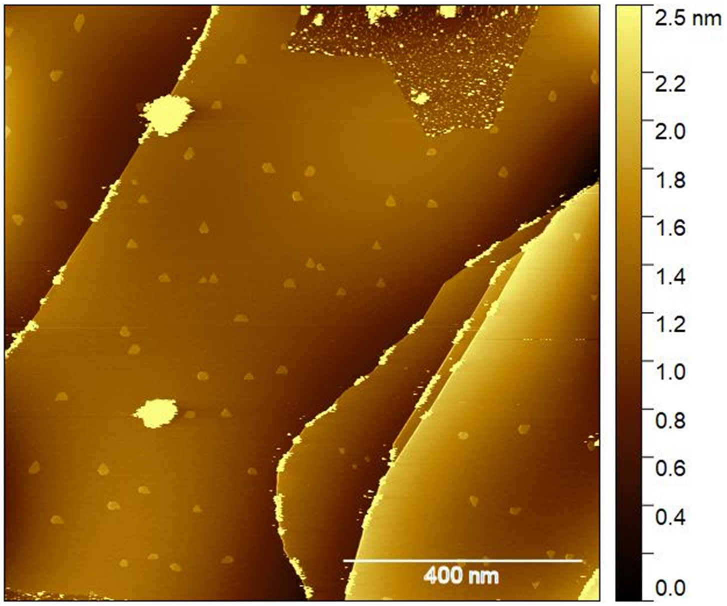

FIG. S6. STM topography of a 1 $\times$ 1 μm^2 area acquired in a ZLG/MLG graphene region, after annealing at 650°C for 5 minutes. The image reveals surface clusters of nanoparticles and a random distribution of intecalated 2D Ni islands within the MLG region, exhibiting lateral sizes ranging from 15 nm to 20 nm. Imaging parameters: V_s= 0.8 V, I_t = 0.70 nA.

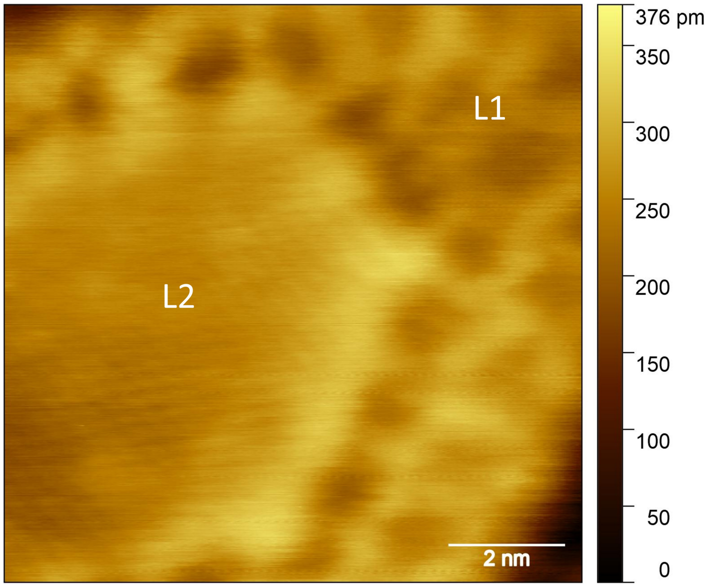

FIG. S7. STM topography of a 10×10 nm^2 region, showing a magnified view of the Ni intercalated area in Figure 5b. Regions L1 and L2 correspond to monolayer and bilayer Ni, respectively. The image reveals an attenuated $6\sqrt{3}$ surface reconstruction in A2 compared to A1. Imaging parameters: V_s= 0.3 V, I_t = 0.25 nA. Annealing conditions: 650°C, 10 minutes.

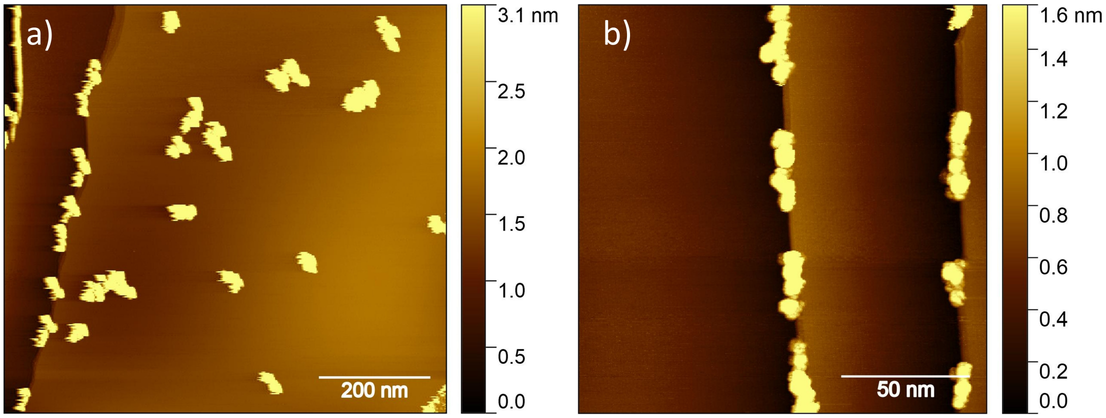

FIG. S8. STM topographies of (a) a 800×800 nm^2 area and (b) a 140×140 nm^2 area in two different MLG region, acquired after annealing the sample at 800 °C for 3 minutes. The images show the presence of Ni NP clusters on the EG surface, but no evidence of intercalated Ni regions. Imaging parameters: (a) V_s= 0.7 V, I_t = 0.60 nA and (b) V_s= 0.5 V, I_t = 0.50 nA.

S4. ADDITIONAL XPS DATA

Figure S9a illustrates how the relative weight of the different XPS C 1s spectral components (Fig. 6b) evolves as a function of temperature, highlighting the thermally induced changes in the chemical environment of carbon.

The Si 2p spectrum of the as-prepared sample (Figure S9b) consists of two components: a main doublet at 101.2 eV, corresponding to bulk SiC, and a weaker component at higher binding energy, typically associated with surface or interface defects.[3] The Si 2p XPS components remain largely unchanged from room temperature through the successive annealing steps. No low-binding energy features associated with nickel silicide formation are detected, indicating that nickel does not intercalate between SiC(0001) and the ZLG.

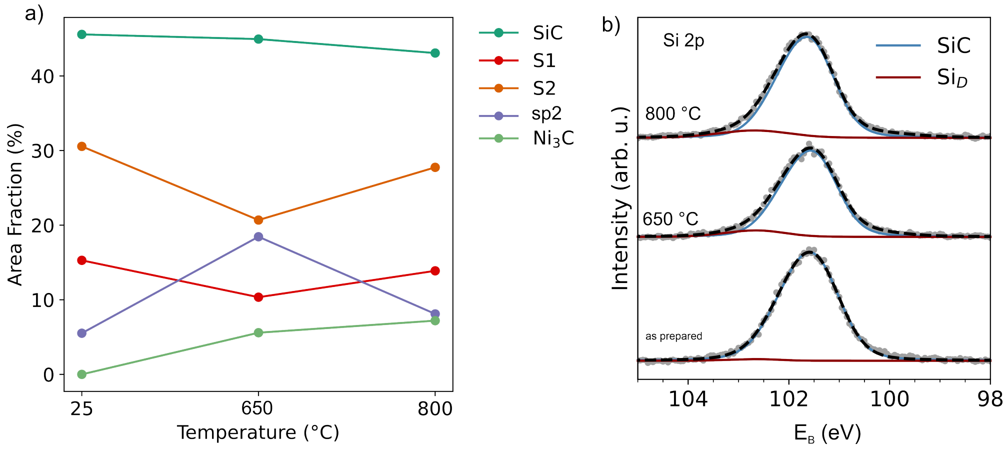

FIG. S9. a) Temperature dependence of the relative intensity of the various components in the C 1s spectrum reported in Fig. 6b of the main text. b) Si 2p core level spectra of the as-prepared sample, and after annealing at 650°C and 800°C.

S5. ARPES MEASUREMENTS

ARPES measurements were carried out to investigate the evolution of the electronic structure of the graphene-2D Ni system. ARPES measurements, shown in Figure S10, reveal significant modifications in the electronic structure upon nickel intercalation. After annealing at 650 °C, the spectra reveal partial Ni intercalation, consistent with the formation of intercalated Ni islands at the ZLG/MLG interface. This partial intercalation results in changes

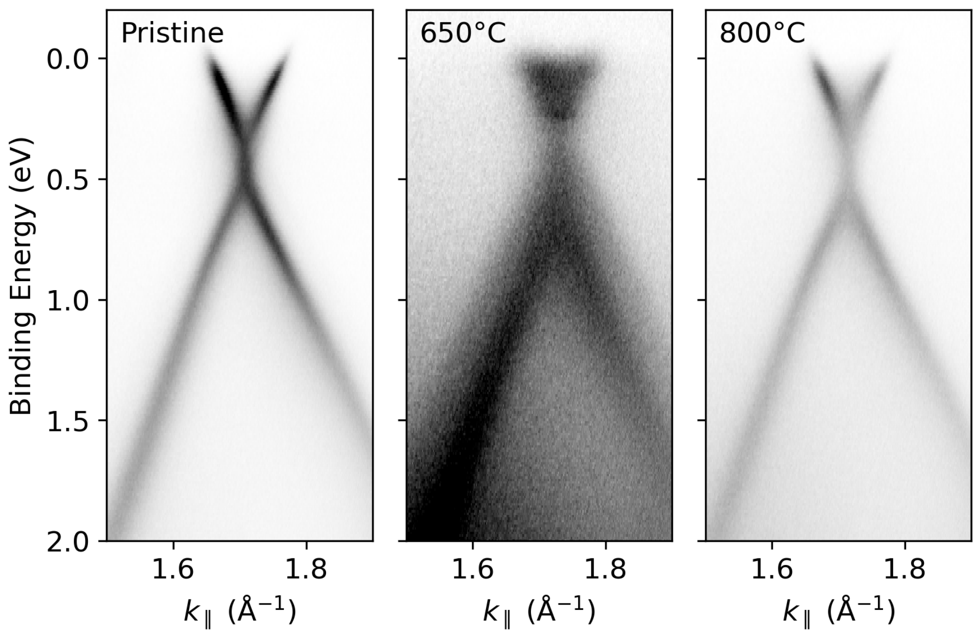

FIG. S10. ARPES spectra around the graphene K-point of the Brillouin zone obtained from (a) the pristine EG sample, and after Ni NPs deposition and annealing at (b) 650 °C and (c) 800 °C.

to the Dirac cone region, suggesting local doping and interaction with Ni. These features are in line with the XPS data, which indicate chemical changes at the graphene/buffer layer interface. Upon further annealing at 800 °C, the ARPES spectra exhibit a restoration of the characteristic linear dispersion of pristine monolayer graphene. This evolution suggests that the system gradually recovers a quasi-pristine graphene configuration, possibly due to redistribution or desorption of Ni, in agreement with the STM and XPS observations.

S6. FURTHER FIRST PRINCIPLES SIMULATIONS

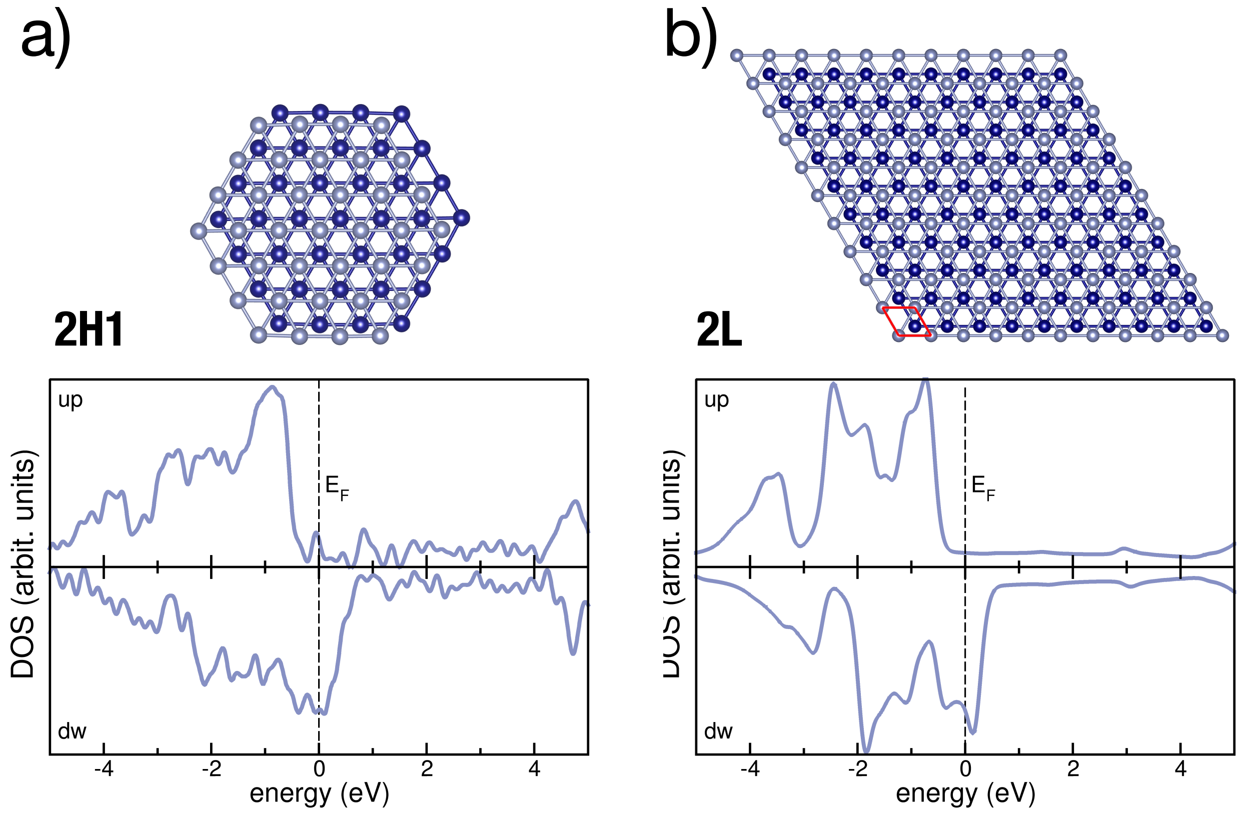

FIG. S11. Top view atomic structure (top panels) and spin-resolved density of states (DOS, bottom panels) of free standing 2D Ni bilayers: a) two smaller hexagonal nanostructures **2H1**, and b) two extended layers **2L**. Vertical alignment follows the planar stack of the Ni surface along the [111] direction. Vertical dashed lines in bottom panels identify the Fermi level (E_F) of each system.

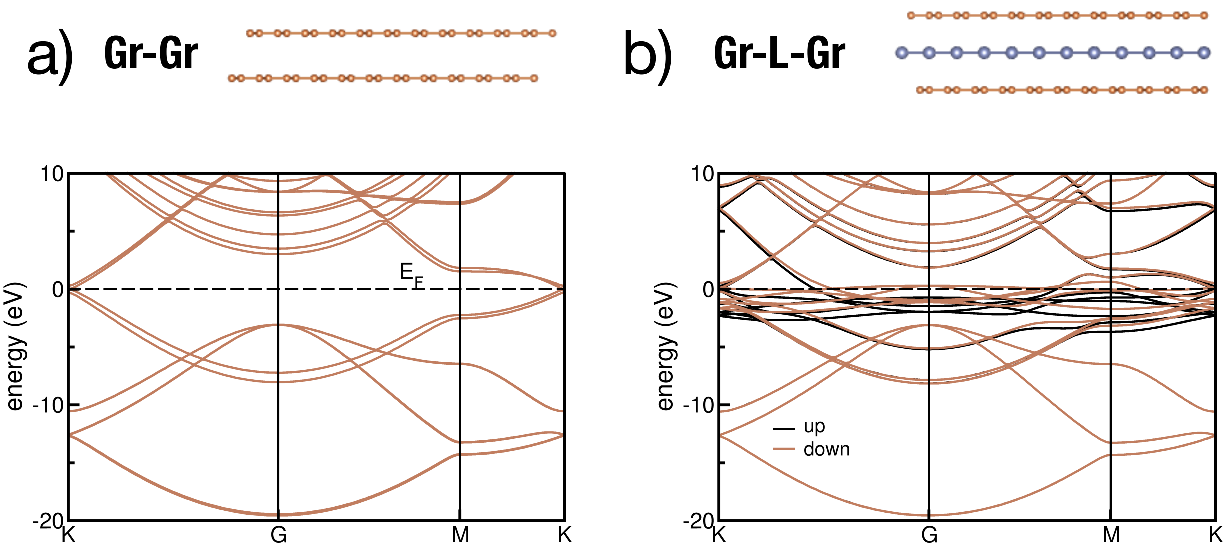

FIG. S12. Side view atomic structure (top panel) and spin-resolved band structure (bottom panel) of a) pristine Gr-Gr bilayer, and b) intercalated Gr-L-Gr layer. Horizontal dashed lines in the bottom panels identify the Fermi level (E_F) of each system.

REFERENCES

[1] M. Kruskopf, K. Pierz, S. Wundrack, R. Stosch, T. Dziomba, C.-C. Kalmbach, A. Müller, J. Baringhaus, C. Tegenkamp, F. J. Ahlers, and H. W. Schumacher, "Epitaxial graphene on SiC: modification of structural and electron transport properties by substrate pretreatment," Journal of Physics: Condensed Matter **27**, 185303 (2015).

[2] Z. H. Ni, W. Chen, X. F. Fan, J. L. Kuo, T. Yu, A. T. S. Wee, and Z. X. Shen, "Raman spectroscopy of epitaxial graphene on a SiC substrate," Physical Review B **77**, 115416 (2008).

[3] K. V. Emtsev, F. Speck, T. Seyller, L. Ley, and J. D. Riley, "Interaction, growth, and ordering of epitaxial graphene on SiC{0001} surfaces: A comparative photoelectron spectroscopy study," Physical Review B **77**, 155303 (2008).
